# Clinical prediction models for progression of chronic kidney disease to end-stage kidney failure under pre-dialysis nephrology care: results from the Chronic Kidney Disease Japan Cohort Study

**DOI:** 10.1007/s10157-018-1621-z

**Published:** 2018-08-01

**Authors:** Takeshi Hasegawa, Kentaro Sakamaki, Fumihiko Koiwa, Tadao Akizawa, Akira Hishida, on behalf of the CKD-JAC Study Investigators

**Affiliations:** 1 Office for Promoting Medical Research, Showa University, 1-5-8 Hatanodai, Shinagawa-ku, Tokyo, 142-8555 Japan; 2 Division of Nephrology (Fujigaoka Hospital), Department of Medicine, Showa University School of Medicine, Yokohama, Japan; 3 Center for Innovative Research for Communities and Clinical Excellence, Fukushima Medical University, Fukushima, Japan; 4 Department of Healthcare Epidemiology, School of Public Health in the Graduate School of Medicine, Kyoto University, Kyoto, Japan; 5 Department of Biostatistics and Bioinformatics, Graduate School of Medicine, The University of Tokyo, Tokyo, Japan; 6 Division of Nephrology, Department of Medicine, Showa University School of Medicine, Tokyo, Japan; 7 Yaizu City Hospital, Shizuoka, Japan

**Keywords:** Clinical prediction modelsm, Chronic kidney disease (CKD), End-stage kidney failure (ESKF), Chronic Kidney Disease Japan Cohort (CKD-JAC) study

## Abstract

**Background:**

Reliable prediction tools are needed to identify patients with chronic kidney disease (CKD) at greater risk of developing end-stage kidney failure (ESKF). We developed and validated clinical prediction models (CPMs) for CKD progression to ESKF under pre-dialysis nephrology care using CKD-Japan Cohort (CKD-JAC) data.

**Methods:**

We prospectively followed up 2034 participants with CKD, defined as an estimated glomerular filtration rate (eGFR) less than 60 mL/min/1.73 m^2^, aged 20–75 years for a mean of 3.15 years. We randomly divided the overall analysis set into development and validation cohorts. In the development cohort, CPMs were developed using Cox proportional hazard regression, and the goodness of fit was evaluated. In the validation cohort, discrimination and calibration of the developed CPMs were evaluated. We also validated developed CPMs in the dataset with the bootstrap method.

**Results:**

ESKF onset was observed in 206 and 216 patients in the development (20.3%) and validation (21.2%) cohorts, respectively. Goodness of fit, discrimination, and calibration were worse for a simple model including age, sex, and eGFR than for a complicated model (plus albuminuria, systolic blood pressure, diabetes, serum albumin, and hemoglobin). The mean absolute difference between the observed and predictive probabilities of ESKF onset at 3 years was lower for the complicated model than for the simple model (1.57 vs. 1.87%).

**Conclusions:**

CPMs employing readily available data could precisely predict progression to ESKF in patients with CKD stage G3a to G5. These developed CPMs may facilitate more appropriate clinical care and shared decision-making between clinicians and patients.

**Electronic supplementary material:**

The online version of this article (10.1007/s10157-018-1621-z) contains supplementary material, which is available to authorized users.

## Introduction

Chronic kidney disease (CKD) is an important prognostic factor for the onset of cardiovascular disease (CVD) [1] and mortality [2] in the general population. The number of adults with CKD is increasing [3] and is expected to reach about 600 million people in the near future, which is equivalent to 1 in 10 adults worldwide [4]. In addition, end-stage kidney failure (ESKF) was estimated to affect 2.6 million people worldwide in 2010, and its prevalence is expected to more than double by 2030 [5]. In Japan, the number of patients with ESKF who are undergoing maintenance dialysis exceeds 310,000 [6], and the social burden (including medical expenses) is increasing annually. If patients with CKD who are in the pre-dialysis period could be referred to nephrologists at the appropriate time by evaluating their risk of developing ESKF, their prognosis could improve, even after the introduction of renal replacement therapy [7]. Therefore, reliable prediction tools to identify patients with CKD who are at a greater risk of developing ESKF are needed for clinical decision-making. However, because the progression of CKD varies widely across individuals [8], it has been difficult to predict a patient’s risk for developing ESKF.

Conventionally, the risk assessment in CKD is stratified by the estimated glomerular filtration rate (eGFR) and proteinuria (or albuminuria), independent of other prognostic factors of ESKF onset [9–11]. This method is recommended in current clinical practice guidelines [12]. Risk assessment tools for ESKF onset have been developed using ad hoc studies of randomized controlled trials (RCTs) in patients with diabetic nephropathy due to type 2 diabetes [13, 14]. A retrospective cohort study reported a risk score to predict the risk of renal replacement therapy in patients with CKD at stages 3 or 4 [15]. Tangri et al. developed and validated clinical prediction models (CPMs) for the progression of CKD at stages 3–5 to ESKF, using data from two Canadian cohorts of patients referred to nephrologists [16]. These CPMs have been validated in a cohort of European patients with CKD [17], as well as patients in other regions [18]. That validation study included Japanese patients. However, they only included a general local population cohort [10, 19] and a local CKD cohort [20].

Therefore, we developed and validated new CPMs for ESKF onset using the Chronic Kidney Disease Japan Cohort study (CKD-JAC) database, which is comprised of representative nephrology clinical center facilities in Japan [21, 22]. The objective of this study was to develop a simple and more accurate CPM that estimates the risk of ESKF onset using primary patient demographic characteristics and laboratory test values, measured in daily clinical practice, in addition to eGFR and proteinuria (albuminuria). Furthermore, we investigated the utility of adding fibroblast growth factor-23 (FGF-23), a strong prognostic factor for both a decline in renal function [23] and mortality [24, 25] in patients with CKD, to this CPM.

## Materials and methods

### Study design, population, and data source

The CKD-Japan Cohort (CKD-JAC) study is a prospective cohort study of pre-dialysis patients with CKD (defined as eGFR less than 60 mL/min/1.73 m^2^), aged 20–75 years, from facilities across Japan. The eGFR for Japanese patients with CKD was calculated using the following formula: eGFR [ml/min/1.73 m^2^] = 194 × age^−0.287^ × serum creatinine^−1.094^ × [0.739 for women] [26]. The CKD-JAC study was conducted at 17 medical institutions that are representative of CKD facilities in Japan. The exclusion criteria of the CKD-JAC study were as follows: (i) patients with polycystic kidney disease, human immunodeficiency virus infection, cirrhosis, active cancer, or cancer treatment within the past 2 years; (ii) transplant recipients and patients who previously underwent chronic dialysis; (iii) pregnant women; and (iv) individuals who refused to provide informed consent. Details of the CKD-JAC study research protocol are described elsewhere [21].

The analyzed population was randomly divided into development and validation cohorts at a ratio of 1:1. The development cohort was used for the development of CPMs, and the validation cohort was used to confirm the validity of developed CPMs. We also validated the CPMs in the dataset using the bootstrap technique with 10,000 re-samples. The findings of this investigation are reported in accordance with the Transparent Reporting of a multivariable prediction model for Individual Prognosis Or Diagnosis (TRIPOD) statement [27].

### Primary outcome

The main outcome measure in this study was ESKF onset, defined as the need for dialysis or preemptive kidney transplantation. Time at risk was defined as the period from study enrollment to ESKF onset, departure from the study (as a result of death prior to ESKF onset, transfer to a non-CKD-JAC facility, or consent withdrawal), or the end of study follow-up.

### Candidate baseline variables for the CPMs

Candidate baseline variables for the CPMs, adopted by reference to previous studies [13–16], consisted of demographic characteristics, including age; sex; physical examination findings, including body mass index (BMI) and systolic blood pressure (SBP); comorbid conditions, including diabetes and hypertension; and laboratory variables, including eGFR, the urinary albumin-creatinine ratio (UACR), serum creatinine, serum sodium, serum albumin, hemoglobin (Hb), serum calcium, serum phosphorus, intact parathyroid hormone (iPTH), and FGF-23. The distributions of UACR, iPTH, and FGF-23 were skewed; therefore, the data were logarithm transformed for analysis.

### Ethical considerations

All procedures in the CKD-JAC study were approved by the institutional review board (IRB) in each facility (IRB approval number 2007578 in Showa University Fujigaoka Hospital) and were performed per the Helsinki Declaration and its later amendments or comparable ethical standards. Informed consent was obtained for each participant in accordance with the requirements of IRB and facility.

### Statistical analyses

Continuous and binary variables were tabulated in the overall analysis set and in each group and are expressed as mean (standard deviation) or median (quartile range), and frequency (proportion), respectively. When data were missing, imputation by statistical methods was not performed. All statistical analyses were performed using SAS version 9.3 and R version 3.2.2. Unless otherwise noted, two-sided *P* values < 0.05 were considered statistically significant.

The CPMs were constructed using Cox proportional hazard modeling in the development cohort and in the overall analysis set. We constructed a total of ten CPMs based on clinical insights and findings from previous studies: model 1 included age and sex; model 2 included model 1 plus eGFR; model 3 included model 2 plus log-UACR; model 4 included model 3 plus SBP; model 5 included model 4 plus diabetes; model 6 included model 5 plus serum albumin; model 7 included model 6 plus Hb; model 8 included model 7 plus log-iPTH; model 9 included model 8 plus log-FGF-23; and model 10 included model 9, without diabetes, plus serum creatinine and serum calcium. Model 10 was based on a statistical forward selection method using all the candidate baseline variables mentioned above. In the variable selection using the forward method, *P* ≤ 0.1 was used as the selection criterion.

The goodness of fit of the CPMs was evaluated in the development cohort and in the overall analysis set using the Akaike Information Criterion (AIC). The discrimination and calibration performance of the developed CPMs were evaluated in the validation cohort and in the data set with bootstrap technique. The integrated area under the curve (AUC), based on time-dependent receiver operating characteristics as proposed by Heagerty and Zheng [28], was used as a C statistic (concordance statistic) for an index of the discrimination performance for survival time. In calculating the integrated AUC, the maximum survival time was set to 3 years. The calibration performance of the CPMs was evaluated using the *χ*^2^ statistic of Nam and D’Agostino [29], in which the observed and predicted risks of ESKF onset at 3 years were compared.

As a sensitivity analysis, we calculated the probability of ESKF onset considering death as a competing risk [30] using a competing risk model assuming proportional hazards to the cause-specific hazard.

## Results

**Fig. 1:**
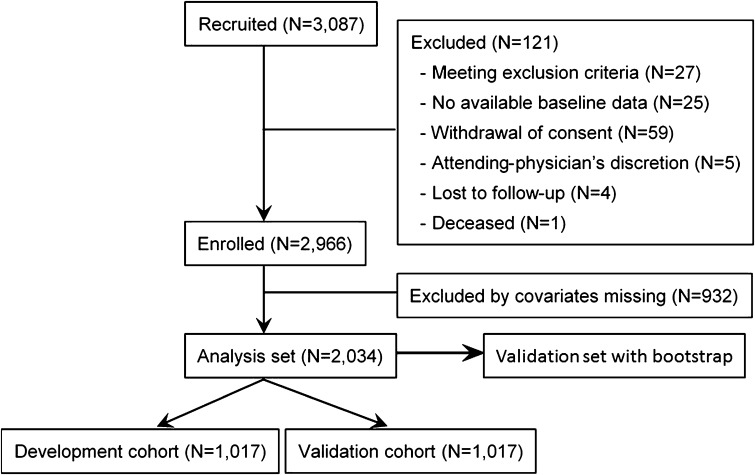
Flow diagram of the analyzed patients

Figure 1 shows the flow of patient selection from the target population of CKD-JAC study to the analysis set of this investigation, along with the reasons for exclusion. The development cohort comprised 1,017 patients, including 206 (20.3%) with ESKF onset (203 with dialysis initiation and 3 with preemptive transplantation), and the validation cohort comprised 1017 patients, including 216 (21.2%) with ESKF onset (213 with dialysis initiation and 3 with preemptive transplantation) (Fig. 1; Table 1). Patient characteristics in the overall analysis set, the development and validation cohorts were almost identical in all variables (Table 1).

**Table 1:** Baseline characteristics and outcomes of the overall analysis set and the development and validation cohorts

Characteristics	Development cohort (*n* = 1017)	Validation cohort (*n* = 1017)
Age, mean (SD), years	60.6 (11.6)	61.1 (11.1)
Male, *n* (%)	642 (63.1)	658 (64.7)
SBP, mean (SD), mmHg	132 (18)	131 (19)
Diabetes, *n* (%)	394 (38.7)	391 (38.5)
Hypertension, *n* (%)	854 (84.0)	855 (84.1)
eGFR, mean (SD), mL/min/1.73 m^2^	28.8 (12.5)	27.5 (12.1)
UACR, median (interquartile range), mg/g	0.48 (0.12–1.32)	0.55 (0.13–1.42)
log-UACR, mean (SD), mg/g	− 0.45 (0.74)	− 0.41 (0.75)
Serum creatinine, mean (SD), mg/dL	2.17 (1.08)	2.26 (1.12)
Serum sodium, mean (SD), mEq/L	140.4 (2.99)	140.4 (3.04)
Serum albumin, mean (SD), g/dL	4.0 (0.4)	4.0 (0.4)
Hemoglobin, mean (SD), g/dL	12.2 (1.9)	12.0 (1.8)
Serum calcium, mean (SD), mg/dL	9.01 (0.52)	8.98 (0.54)
Serum phosphorus, mean (SD), mg/dL	3.53 (0.69)	3.53 (0.71)
iPTH, median (interquartile range), pg/mL	78 (52–124)	84 (58–132)
log-iPTH, mean (SD), pg/mL	1.92 (0.29)	1.95 (0.3)
FGF-23, median (interquartile range), pg/mL	57.7 (40.2–89.8)	58.4 (42.0–98.7)
log-FGF-23, mean (SD), pg/mL	1.84 (0.39)	1.86 (0.40)
Outcomes		
Observation time, years	3.17 (1.17)	3.14 (1.18)
Death, *n* (%)	27 (2.6)	30 (2.9)
ESKF onset, *n* (%)	206 (20.3)	216 (21.2)
Dialysis	203 (20.0)	213 (20.9)
Transplantation	3 (0.3)	3 (0.3)

*SD* standard deviation, *SBP* systolic blood pressure, *eGFR* estimated glomerular filtration rate, *UACR* urine-albumin to creatinine ratio, *iPTH* intact parathyroid hormone, *FGF-23* fibroblast growth factor 23, *ESKF* end-stage kidney failure

Tables 2 and 3 show the hazard ratios (HRs) and AIC for each CPM in the development cohort using the random split method and in the overall analysis set, respectively. Model 2, with age, sex, and eGFR, had a larger AIC (2,350.1) compared to that for model 7 (model 1 plus SBP, diabetes, log-UACR, serum albumin, and Hb) and model 9 (model 7 plus log-iPTH and log-FGF-23), which had AICs of 2,216.3 and 2,211.6, respectively. Model 10, constructed using the forward selection method (model 9 without diabetes plus serum creatinine and serum calcium), had the lowest (best) AIC (2,203.9) in the development cohort (Table 2). Table 3 shows similar findings in the rank order of AICs in the overall analysis set. The lowest (best) AIC presented in the model 10 came from the forward selection stepwise method (model 8 without diabetes plus hypertension, serum calcium, serum phosphorus, and log-FGF-23).

**Table 2:** Hazard ratios and goodness of fit for the CPMs in the development cohort using the random split method

Variables	Model 1	Model 2	Model 3	Model 4	Model 5	Model 6	Model 7	Model 8	Model 9	Model 10
Age, HR (95% CI) per 10 years	1.00 (0.88–1.12)	0.82 (0.72–0.93)	0.86 (0.76–0.98)	0.84 (0.74–0.96)	0.82 (0.72–0.93)	0.78 (0.69–0.89)	0.76 (0.67–0.86)	0.78 (0.68–0.89)	0.78 (0.69–0.89)	0.83 (0.73–0.95)
Sex, HR (95% CI) male vs. female	1.67 (1.22–2.27)	2.52 (1.84–3.44)	2.15 (1.57–2.94)	2.15 (1.57–2.94)	2.11 (1.53–2.87)	2.07 (1.51–2.84)	2.43 (1.77–3.35)	2.42 (1.76–3.33)	2.38 (1.73–3.28)	1.53 (1.02–2.28)
SBP, HR (95% CI) per 10 mmHg				1.12 (1.03–1.20)	1.12 (1.04–1.21)	1.13 (1.05–1.22)	1.12 (1.04–1.21)	1.12 (1.04–1.21)	1.13 (1.05–1.21)	1.14 (1.06–1.23)
Diabetes, HR (95% CI) yes vs. no					1.53 (1.16–2.04)	1.46 (1.10–1.94)	1.38 (1.04–1.85)	1.40 (1.05–1.86)	1.41 (1.06–1.87)	
eGFR, HR (95% CI) per 1 mL/min/1.73 m^2^		0.85 (0.83–0.87)	0.92 (0.91–0.93)	0.92 (0.91–0.93)	0.92 (0.91–0.93)	0.92 (0.91–0.93)	0.93 (0.92–0.94)	0.64 (0.46–0.91)	0.88 (0.86–0.91)	0.97 (0.95–0.99)
log-UACR, HR (95% CI) per 1 mg/g			3.86 (2.84–5.25)	3.48 (2.54–4.78)	3.15 (2.30–4.32)	2.36 (1.69–3.28)	2.85 (2.00-4.04)	2.77 (1.95–3.93)	2.83 (1.98–4.03)	2.78 (1.94–3.99)
Serum creatinine, HR (95% CI), per 1 mg/dL										1.65 (1.22–2.22)
Serum albumin, HR (95% CI), per 1 g/dL						0.51 (0.37–0.71)	0.67 (0.47–0.94)	0.64 (0.46–0.91)	0.64 (0.45–0.89)	0.70 (0.48–1.01)
Hemoglobin, HR (95% CI) per 1 g/dL							0.79 (0.71–0.87)	0.79 (0.71–0.88)	0.81 (0.73–0.90)	0.81 (0.73–0.90)
Serum calcium, HR (95% CI), per 1 mg/dL										0.71 (0.53–0.96)
log-iPTH, HR (95% CI) per 1 pg/mL								1.66 (0.90–3.06)	1.74 (0.96–3.18)	
log-FGF-23, HR (95% CI) per 1 pg/mL									1.53 (1.11–2.13)	1.51 (1.07–2.12)
AIC	2730.1	2350.1	2261.9	2256.5	2249.8	2236.8	2216.3	2215.6	2211.6	2203.9

*CPMs* clinical prediction models, *HR* hazard ratio, *CI* confidence interval, *SBP* systolic blood pressure, *eGFR* estimated glomerular filtration rate, *UACR* urine-albumin to creatinine ratio, *iPTH* intact parathyroid hormone, *FGF23* fibroblast growth factor 23, *ESKF* end-stage kidney failure, *AIC* Akaike Information Criterion (lower values for AIC represent better models)

**Table 3:** Hazard ratios and goodness of fit for the CPMs in the overall analysis set

Variables	Model 1	Model 2	Model 3	Model 4	Model 5	Model 6	Model 7	Model 8	Model 9	Model 10
Age, HR (95% CI) per 10 years	1.00 (0.92–1.09	0.88 (0.80–0.96)	0.92 (0.84–1.01)	0.91 (0.83–1.00)	0.90 (0.82–0.99)	0.88 (0.80–0.96)	0.83 (0.76–0.92)	0.85 (0.77–0.93)	0.86 (0.78–0.94)	0.85 (0.78–0.94)
Sex, HR (95% CI) male vs. female	1.78 (1.43–2.21)	1.12 (0.90–1.40)	1.06 (0.85–1.32)	1.05 (0.84–1.30)	1.03 (0.82–1.28)	1.00 (0.80–1.25)	1.26 (1.00-1.59)	1.32 (1.04–1.67)	1.33 (1.05–1.68)	1.41 (1.11–1.79)
SBP, HR (95% CI) per 10 mmHg				1.09 (1.03–1.16)	1.09 (1.03–1.15)	1.10 (1.04–1.16)	1.10 (1.04–1.16)	1.10 (1.04–1.15)	1.10 (1.04–1.16)	1.09 (1.04–1.15)
Diabetes, HR (95% CI) yes vs. no					1.19 (0.97–1.45)	1.13 (0.92–1.38)	1.10 (0.90–1.35)	1.10 (0.90–1.34)	1.09 (0.89–1.33)	
Hypertension, HR (95% CI) yes vs. no										1.41 (1.00–1.99)
eGFR, HR (95% CI) per 1 mL/min/1.73 m^2^		0.86 (0.85–0.87)	0.87 (0.86–0.88)	0.87 (0.86–0.88)	0.87 (0.86–0.88)	0.87 (0.86–0.88)	0.88 (0.87–0.90)	0.89 (0.88–0.90)	0.89 (0.88–0.91)	0.90 (0.89–0.91)
log-UACR, HR (95% CI) per 1 mg/g			4.39 (3.50–5.50)	4.04 (3.21–5.08)	3.84 (3.04–4.86)	2.96 (2.31–3.80)	3.42 (2.65–4.42)	3.34 (2.58–4.33)	3.36 (2.60–4.35)	3.59 (2.85–4.52)
Serum sodium, HR (95% CI) per 1 mEq/L										0.97 (0.93-1.00)
Serum albumin, HR (95% CI) per 1 g/dL						0.58 (0.47–0.74)	0.75 (0.60–0.95)	0.74 (0.59–0.92)	0.74 (0.59–0.93)	0.70 (0.48–1.01)
Hemoglobin, HR (95% CI) per 1 g/dL							0.77 (0.72–0.84)	0.78 (0.72–0.84)	0.79 (0.73–0.85)	0.81 (0.75–0.87)
Serum calcium, HR (95% CI) per 1 mg/dL										0.73 (0.61–0.88)
Serum phosphorus, HR (95% CI), per 1 mg/dL										1.30 (1.12–1.51)
log-iPTH, HR (95% CI) per 1 pg/mL								1.48 (0.97–2.26)	1.49 (0.96–2.26)	
log-FGF-23, HR (95% CI) per 1 pg/mL									1.43 (1.13–1.81)	1.46 (1.14–1.85)
AIC	6078.5	5363.5	5157.7	5150.2	5149.4	5130.5	5087.4	5086.0	5079.9	5040.1

*CPMs* clinical prediction models, *HR* hazard ratio, *CI* confidence interval, *SBP* systolic blood pressure, *eGFR* estimated glomerular filtration rate, *UACR* urine-albumin to creatinine ratio, *iPTH* intact parathyroid hormone, *FGF23* fibroblast growth factor 23, *ESKF* end-stage kidney failure, *AIC* Akaike Information Criterion (lower values for AIC represent better models)

The C statistics reflecting the discrimination ability in the validation cohort using the random split method and in the validation dataset using the bootstrap method for the developed CPMs are shown in Supplementary Tables 1A and 1B, respectively. Compared to that in model 2, the C statistic was improved in model 7 (0.837 vs. 0.875). However, there was no further improvement with the inclusion of log-iPTH and log-FGF-23 (model 9) or with stepwise forward selection (model 10) in both the validation cohort using the random split method and validation dataset using the bootstrap method. The calibration performance of CPMs in the validation cohort and validation dataset, as indicated by the *χ*^2^ statistic of Nam and D’Agostino, improved with the successive inclusion of variables in models 2, 7, and 9, but did not further improve in model 10 (Supplementary Tables 1A and 1B).

**Fig. 2:**
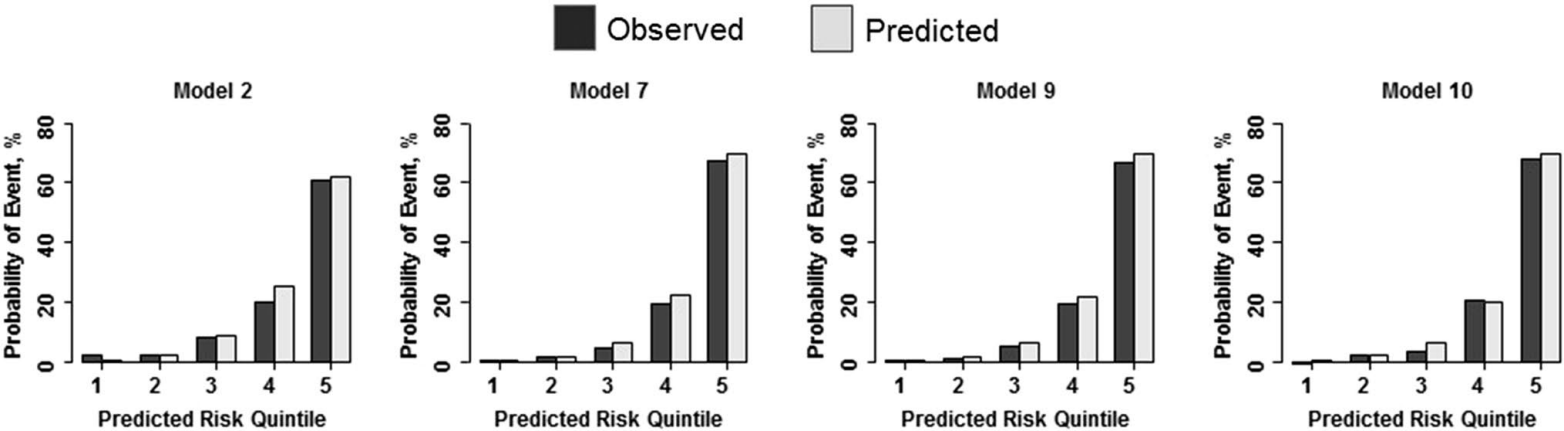
Observed vs. predicted probability of ESKF at 3 years in the validation cohort. Mean predicted probability of ESKF onset at 3 years for quintiles 1 through 5 corresponds to 0.5, 2.3, 8.7, 25.2, and 62.1%, respectively, for model 1; 0.2, 1.5, 6.2, 22.1, and 69.3%, respectively, for model 2; 0.2, 1.5, 6.1, 21.7, and 69.5%, respectively, for model 3; and 0.4, 2.0, 6.3, 20.0, and 69.5%, respectively, for model 4. Abbreviations: ESKF, end-stage kidney failure

We estimated the probability of ESKF onset at 3 years without (Observed: crude incidence rate) and with the CPMs (Predicted: predicted rate) in the validation cohort (Fig. 2). Patients were stratified by quintiles of predicted probabilities. The mean absolute difference between the observed and predicted probabilities over the quintiles of risk for ESKF onset at 3 years was the lowest (best) for model 10, followed by models 9, 7, and 2 (1.10 vs. 1.42%, 1.57, and 1.87%, respectively).

Survival analyses considering death as a competing risk did not differ from the observed Kaplan–Meier method estimate or the Cox proportional hazards model. Supplementary Fig. 1a shows the results of Kaplan–Meier method without the consideration of death as a competing risk, and cumulative incidence with the consideration of death as a competing risk. Similarly, Supplementary Fig. 1b shows the probabilities of ESKF onset using the Cox proportional hazards model without consideration of death as a competing risk, and a competing risk model assuming proportional hazards to the cause-specific hazard.

Supplementary Table 2 describes the demographic characteristics, physical examination findings, comorbid conditions, and laboratory findings in two hypothetical patients with the same eGFR (20 mL/min/1.73 m^2^), as well as their probability of ESKF onset using the CPMs (models 2 and 7). The predicted probabilities of ESKF onset at 3 years using model 2 (eGFR, age, and sex) were 10.9 and 29.8% in patients A and B, respectively. The additional inclusion of SBP, diabetes, log-UACR, serum albumin, and Hb (model 7) resulted in substantially different probabilities, with the predicted probability of ESKF onset at 3 years reduced by 7.6% in patient A and increased by 11.9% in patient B.

## Discussion

We developed and validated a new CPM that accurately predicts the 3-year probability of ESKF onset in patients with CKD who are receiving pre-dialysis nephrology care. The AIC, which represents the goodness of fit of the CPM, was worse for model 2 (the simpler model, comprised of age, sex, and eGFR) than for model 7 (including model 2 plus SBP, diabetes, log-UACR, serum albumin, and Hb). Model 7 improved the integrated AUC for discrimination and the Nam and D’Agostino statistics for calibration compared to model 2. In addition, model 9 (adding log-iPTH and log-FGF-23 to model 7) had a better estimation of the Nam and D’Agostino statistic than model 7. Furthermore, the mean absolute difference between the observed and predicted probabilities of ESKF onset at 3 years was lower for model 9 (1.42%) than for model 2 (1.87%) or model 7 (1.57%). As shown in Supplementary Table 2, the predicted probability of ESKF onset differed substantially between the simpler (model 2) and complicated (model 7) models.

The CPMs developed and validated in the present investigation were based on demographic characteristics, comorbidities, and laboratory variables collected routinely in daily clinical practice. Similar to findings of previous studies, younger age [15], male sex [31], lower eGFR and higher albuminuria at baseline [10, 32], and lower serum albumin [13] were related to a faster progression to ESKF (i.e., they predicted earlier ESKF onset). All these variables were also included in the CPMs reported by Tangri et al. [16].

FGF-23 has been associated with the risk of ESKF onset, not only in CKD patients [33], but also in a community-based population [34]. However, few studies have evaluated the utility of FGF-23 to incrementally improve CPMs for estimating the ESKF onset. In the present study, the addition of log-FGF-23 to the developed CPMs did not further improve their discrimination ability.

The current study has several additional clinical implications and significant findings. The developed CPMs, based on the CKD-JAC cohort, may facilitate more appropriate clinical decision-making for clinicians and patients than that provided by the CKD stages recommended in the existing clinical guidelines, which are based on eGFR and proteinuria (albuminuria) alone [12]. Applying the CPMs developed and validated in the present investigation could also help to provide individual CKD patients with the necessary knowledge and interventions (e.g., dialysis modality education, vascular or peritoneal access creation, and preemptive kidney transplantation) at the optimal time. The developed CPMs can also be used to estimate the incidence of ESKF in future clinical trials and enhance the statistical power by selecting high-risk patients.

The risk of ESKF onset among patients with CKD varies with not only the patient’s background, demographic, comorbidities, and laboratory values, but also their risk of death before ESKF onset. In other words, death could be a competing risk factor for ESKF onset. However, in the present study, no significant differences in the results were observed in the sensitivity analyses with death considered as a competing risk.

One strength of the present analysis is its application of data that are readily available and routinely collected in clinical settings, making the CPMs highly practical. Furthermore, the CPMs developed and validated in the current study had equal or better capacities for calibration than the existing CPMs widely used in multiple cohorts [16]. In the present study, the best *χ*^2^ statistic of Nam and D’Agostino and the mean absolute difference between the observed and predicted probabilities of the ESKF onset were 3.27 and 1.10%, respectively, whereas they were 19.0 and 1.90%, respectively, in a previous report [16]. However, we could not directly evaluate these existing CPMs [15] in our cohort. The CPM by Tangri et al. [15] included serum bicarbonate level as one of the candidate predictors, yet the measurement of bicarbonate is not a common practice in Japan and a large number of the data points were missing in the CKD-JAC cohort.

The present study has some limitations. First, candidate variables for the CPMs were determined only once at baseline. This practice could fail to fully explain the measurement variability in CKD patients and their evolving risk of ESKF onset over time, as both a decline in eGFR [35] and an increase in albuminuria [36] are strongly and consistently associated with the risk of ESKF onset. Second, since the patients enrolled in the CKD-JAC cohort were diagnosed with CKD at stage G3 or higher (eGFR less than 60 mL/min/1.73 m^2^), it is not possible to extrapolate the CPMs developed and validated in the current investigation to early-stage CKD, i.e., stages G1 and G2 (eGFR 60−90 mL/min/1.73 m^2^). Third, we could not validate the developed CPMs in an external dataset, which would be ideal for both internal and external validation. Fourth, although the CKD population is becoming older worldwide, the enrolled patients aged 20 to 75 years in this study were relatively young. Therefore, other validation cohorts are needed to clarify whether these CPMs are applicable to patients older than this age group. Finally, a selection bias cannot be ruled out, as patients were mostly enrolled at larger hospitals that provide pre-dialysis nephrology care. This bias limits the generalizability of the present findings to patients with CKD who have not been referred to a nephrologist.

In conclusion, the new CPMs developed and validated in the present study employ readily available data routinely collected in clinical settings can accurately predict progression to ESKF in patients with CKD at stages G3a to G5. The use of the developed CPMs may facilitate more appropriate clinical care and shared decision-making among clinicians and patients. The addition of FGF-23 level to the CPMs did not further improve their capacities for discrimination. Further investigations are needed to perform an external validation in various CKD populations, including patients without pre-dialysis nephrology care.

## Electronic supplementary material

Below is the link to the electronic supplementary material.

Results from the sensitivity analyses considering death as a competing risk. (a) Comparison of the cumulative incidence of ESKF with or without considering death as a competing risk using the Kaplan-Meier method. (b) Comparison of the predicted probability of ESKF with or without considering death as a competing risk using a Cox proportional hazards model. The bar graph expresses the mean probability of ESKF onset for each patient at 0.5, 1, 1.5, 2, 2.5, and 3 years. Abbreviations: ESKF, end-stage kidney failure (TIF 100 KB)

Supplementary material 2 (DOCX 22 KB)

Supplementary material 3 (DOCX 15 KB)

Supplementary material 4 (DOCX 14 KB)
