# Supplementary figures and images for: Clinical prediction models for progression of chronic kidney disease to end-stage kidney failure under pre-dialysis nephrology care: results from the Chronic Kidney Disease Japan Cohort Study
Source: Clin Exp Nephrol. 2018 Aug 1;23(2):189–98. doi: 10.1007/s10157-018-1621-z (PMC6510807; doi:10.1007/s10157-018-1621-z)

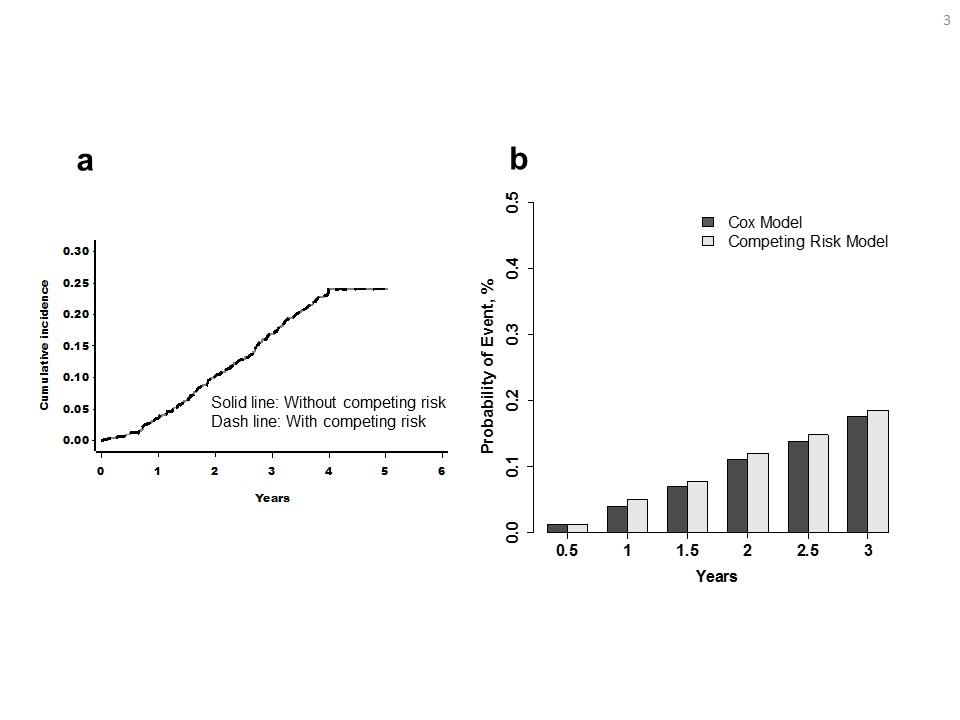

Supplement: Supplementary file 1 — Results from the sensitivity analyses considering death as a competing risk. (a) Comparison of the cumulative incidence of ESKF with or without considering death as a competing risk using the Kaplan-Meier method. (b) Comparison of the predicted probability of ESKF with or without considering death as a competing risk using a Cox proportional hazards model. The bar graph expresses the mean probability of ESKF onset for each patient at 0.5, 1, 1.5, 2, 2.5, and 3 years. Abbreviations: ESKF, end-stage kidney failure (TIF 100 KB) [file 10157_2018_1621_MOESM1_ESM.tif]
