# Supplementary material for: Clinical prediction models for progression of chronic kidney disease to end-stage kidney failure under pre-dialysis nephrology care: results from the Chronic Kidney Disease Japan Cohort Study
Source: Clin Exp Nephrol. 2018 Aug 1;23(2):189–98. doi: 10.1007/s10157-018-1621-z (PMC6510807; doi:10.1007/s10157-018-1621-z)
Supplement: Supplementary file 2 — Supplementary material 2 (DOCX 22 KB) [file 10157_2018_1621_MOESM2_ESM.docx]

**Supplementary Table 1A.** Discrimination and calibration for models in the validation cohort using the random split method

| **Variables** | **Model 1** | **Model 2** | **Model 3** | **Model 4** | **Model 5** | **Model 6** | **Model 7** | **Model 8** | **Model 9** | **Model 10** |
| --- | --- | --- | --- | --- | --- | --- | --- | --- | --- | --- |
| Integrated AUC | 0.568 | 0.837 | 0.873 | 0.873 | 0.869 | 0.868 | 0.875 | 0.875 | 0.875 | 0.875 |
| Nam and D’Agostino statistics | 7.48 | 16.0 | 6.03 | 6.61 | 5.41 | 6.63 | 4.61 | 4.20 | 3.27 | 4.38 |
| Model 1: Age and sex included | | | | | | | | | | |
| Model 2: Model 1 plus eGFR included |  |  |  |  |  |  |  |  |  |  |
| Model 3: Model 2 plus log UACR included |  |  |  |  |  |  |  |  |  |  |
| Model 4: Model 3 plus SBP included |  |  |  |  |  |  |  |  |  |  |
| Model 5: Model 4 plus diabetes included |  |  |  |  |  |  |  |  |  |  |
| Model 6: Model 5 plus serum albumin included |  |  |  |  |  |  |  |  |  |  |
| Model 7: Model 6 plus hemoglobin included |  |  |  |  |  |  |  |  |  |  |
| Model 8: Model 7 plus log iPTH included |  |  |  |  |  |  |  |  |  |  |
| Model 9: Model 8 plus log FGF-23 included |  |  |  |  |  |  |  |  |  |  |
| Model 10 (constructed by stepwise forward selection method using p value less equal 0.1): Age, sex, eGFR, log UACR, SBP, serum creatinine, serum albumin, hemoglobin, serum calcium, and log FGF-23 included | | | | | | | | | | |
| Abbreviations: AUC, area under the curve; eGFR, estimated glomerular filtration rate; UACR, urine-albumin to creatinine ratio; SBP, systolic blood pressure; iPTH, intact parathyroid hormone; FGF-23, fibroblast growth factor 23. | | | | | | | | | | |
