# Supplementary material for: Clinical prediction models for progression of chronic kidney disease to end-stage kidney failure under pre-dialysis nephrology care: results from the Chronic Kidney Disease Japan Cohort Study
Source: Clin Exp Nephrol. 2018 Aug 1;23(2):189–98. doi: 10.1007/s10157-018-1621-z (PMC6510807; doi:10.1007/s10157-018-1621-z)
Supplement: Supplementary file 3 — Supplementary material 3 (DOCX 15 KB) [file 10157_2018_1621_MOESM3_ESM.docx]

**Supplementary Table 1B.** Discrimination and calibration for models in the validation dataset using the bootstrap method

| **Variables** | **Model 1** | **Model 2** | **Model 3** | **Model 4** | **Model 5** | **Model 6** | **Model 7** | **Model 8** | **Model 9** | **Model 10** |
| --- | --- | --- | --- | --- | --- | --- | --- | --- | --- | --- |
| Integrated AUC, Mean (SD) | 0.566  (0.002) | 0.845  (0) | 0.876  (0.001) | 0.878  (0.001) | 0.878  (0.001) | 0.878  (0.001) | 0.885  (0.001) | 0.885  (0.001) | 0.886  (0.001) | 0.885  (0.003) |
| Nam and D’Agostino statistics, Mean (SD) | 5.40  (2.13) | 16.9  (8.00) | 8.54  (4.62) | 8.45  (4.62) | 8.79  (4.4) | 7.88  (3.83) | 7.12  (4.04) | 6.54  (3.92) | 5.77  (3.83) | 6.92  (3.22) |
| Model 1: Age and sex included | | | | | | | | | | |
| Model 2: Model 1 plus eGFR included |  |  |  |  |  |  |  |  |  |  |
| Model 3: Model 2 plus log UACR included |  |  |  |  |  |  |  |  |  |  |
| Model 4: Model 3 plus SBP included |  |  |  |  |  |  |  |  |  |  |
| Model 5: Model 4 plus diabetes included |  |  |  |  |  |  |  |  |  |  |
| Model 6: Model 5 plus serum albumin included |  |  |  |  |  |  |  |  |  |  |
| Model 7: Model 6 plus hemoglobin included |  |  |  |  |  |  |  |  |  |  |
| Model 8: Model 7 plus log iPTH included |  |  |  |  |  |  |  |  |  |  |
| Model 9: Model 8 plus log FGF-23 included |  |  |  |  |  |  |  |  |  |  |
| Model 10 (constructed by stepwise forward selection method using p value less equal 0.1): Age, sex, eGFR, log UACR, SBP, hypertension, serum sodium, serum albumin, hemoglobin, serum calcium, serum phosphorus, and log FGF-23 included | | | | | | | | | | |
| Abbreviations: AUC, area under the curve; eGFR, estimated glomerular filtration rate; UACR, urine-albumin to creatinine ratio; SBP, systolic blood pressure; iPTH, intact parathyroid hormone; FGF-23, fibroblast growth factor 23. | | | | | | | | | | |
