# Supplementary material for: Clinical prediction models for progression of chronic kidney disease to end-stage kidney failure under pre-dialysis nephrology care: results from the Chronic Kidney Disease Japan Cohort Study
Source: Clin Exp Nephrol. 2018 Aug 1;23(2):189–98. doi: 10.1007/s10157-018-1621-z (PMC6510807; doi:10.1007/s10157-018-1621-z)
Supplement: Supplementary file 4 — Supplementary material 4 (DOCX 14 KB) [file 10157_2018_1621_MOESM4_ESM.docx]

**Supplementary Table 2.** Predicted probability of ESKF at 3 years using the CPMs for hypothetical patients

|  | eGFR^*#^mL/min/1.73 m^2^ | Age^*#^ years | Sex^*#^ | SBP^#^ mmHg | Diabetes^#^ | UACR^#^ mg/g | Serum albumin^#^ g/dL | Hemoglobin^#^ g/dL | **Predicted probability of ESKF at 3 years, %** | |
| --- | --- | --- | --- | --- | --- | --- | --- | --- | --- | --- |
|  |  |  |  |  |  |  |  |  | **Model 2^*^** | **Model 7^#^** |
| Patient A | 20 | 70 | Female | 130 | No | 50 | 4.0 | 10.0 | **10.9** | **3.3** |
| Patient B | 20 | 60 | Male | 150 | Yes | 500 | 3.5 | 11.0 | **29.8** | **41.7** |
| Abbreviations: ESKF, end-stage kidney failure; CPMs, clinical prediction models; eGFR, estimated glomerular filtration rate; SBP, systolic blood pressure; UACR, urine-albumin to creatinine ratio.  ^*^Model 2: Age and sex plus eGFR included.  ^#^Model 7: Model 2 plus log UACR, SBP, diabetes, serum albumin, hemoglobin, and log iPTH included. | | | | | | | | | | |
